# Probing Spin Accumulation induced Magnetocapacitance in a Single Electron Transistor

**DOI:** 10.1038/srep13704

**Published:** 2015-09-08

**Authors:** Teik-Hui Lee, Chii-Dong Chen

**Affiliations:** 1 Department of Physics, National Taiwan University, Taipei 106, Taiwan; 2 Nano Science and Technology Program, Taiwan International Graduate Program, Academia Sinica, Taipei 115, Taiwan; 3 Institute of Physics, Academia Sinica, Taipei 115, Taiwan

## Abstract

The interplay between spin and charge in solids is currently among the most discussed topics in condensed matter physics. Such interplay gives rise to magneto-electric coupling, which in the case of solids was named magneto-electric effect, as predicted by Curie on the basis of symmetry considerations. This effect enables the manipulation of magnetization using electrical field or, conversely, the manipulation of electrical polarization by magnetic field. The latter is known as the magnetocapacitance effect. Here, we show that non-equilibrium spin accumulation can induce tunnel magnetocapacitance through the formation of a tiny charge dipole. This dipole can effectively give rise to an additional serial capacitance, which represents an extra charging energy that the tunneling electrons would encounter. In the sequential tunneling regime, this extra energy can be understood as the energy required for a single spin to flip. A ferromagnetic single-electron-transistor with tunable magnetic configuration is utilized to demonstrate the proposed mechanism. It is found that the extra threshold energy is experienced only by electrons entering the islands, bringing about asymmetry in the measured Coulomb diamond. This asymmetry is an unambiguous evidence of spin accumulation induced tunnel magnetocapacitance, and the measured magnetocapacitance value is as high as 40%.

The study on magnetocapacitance has been motivated by its fundamental interest and high practical importance, and that stimulated the revival of attention in this field about one decade ago^1^. The potential applications include magnetic-field sensors^2^ and electric-write magnetic-read memory devices^3^. Experimentally, two types of magnetocapacitance were revealed: The first type is magnetic-field strength dependent, and is typically found in multiferroic materials with perovskite superlattice structure such as BiMnO_3_^4^ and La_0.875_Sr_0.125_MnO_3_^5^. The second type, also known as tunnel magnetocapacitance (TMC), is found in magnetic tunnel junctions consisting of AlO_x_^6^,^7^ and MgO^8^ barriers. For the latter, the magnetocapacitance varies with magnetization alignment configuration of the two constituent ferromagnetic electrodes, and the charge screening length at the interface of the tunnel barrier was predicted to be elongated by the spin-dependent diffusion constants^9^,^10^. This extended screening length was then used to deduce the effective capacitance that departed from the geometrical capacitance. However, this simple approach may overlook the details of the charge distribution, which can be important for the determination of effective capacitance value. Therefore, a precise calculation of charge distribution on microscopic scale is desirable. Furthermore, because the screening length cannot be directly measured, the physical picture of the microscopic origin behind TMC is hardly appreciated.

Here, we present a clear microscopic mechanism for TMC. When magnetic tunnel junction is aligned in anti-parallel (AP), accumulation of minority spins and depletion of majority spins take place at the interfaces^11^,^12^, and this induces a difference in the interfacial Fermi levels of the majority and minority spins^13^. Thus, the spins diffuse from the interface with different diffusion lengths, and that gives rise to a difference between majority/minority spin density distributions. As a result, there are distinct, adjacent accumulation zone and depletion zone in the total charge density distribution that form a tiny charge dipole. This charge dipole, rather than single exponential charge decay, corresponds to an extra serial capacitance that is responsible for the measured TMC. The extra serial capacitance poses an extra energy required for spins to flip when electrons tunnel through an AP-aligned magnetic junction, giving rise to the observed TMC.

To determine junction capacitance, AC impedance method is typically used. However it may involve complex frequency-dependent dispersion behavior^14^. In our study, we get rid of this complication by incorporating magnetic tunnel junction into a single-electron-transistor (SET). The junction capacitance is accurately determined by utilizing the charging effect. Specifically, the junction capacitance in various magnetization alignment configurations were derived from the slopes of the Coulomb diamond^15^. With this, electrons that tunnel into the island through an AP-aligned junction always experience TMC. This TMC is unambiguously confirmed by the observed asymmetric Coulomb diamond in an anti-parallel (AP) aligned ferromagnetic single-electron-transistor. In the sequential tunneling regime, a single spin flip costs an extra energy originated from the TMC.

## Methods & Results

**Figure 1:**
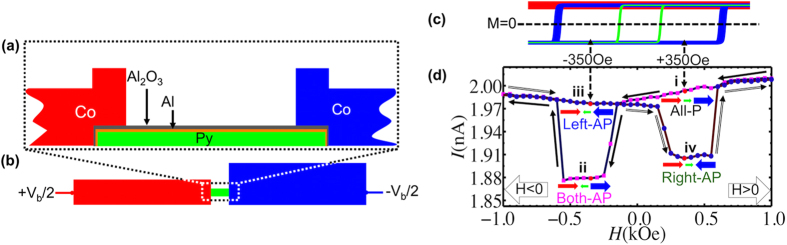
Fabrication and measurement process. (**a**,**b**) schematic drawings of the device in cross-section view and top view. Throughout this paper, we use blue, green, and red to indicate right electrode, center island, and left electrode, respectively. The magnetic field is applied parallel to the long-axis of the two electrodes. The right electrode is made wider to decreases the coercivity. (**c**) Magnetization curves for right electrode (blue), center island (green), and left electrode (red). Note that the curve for left electrode shows no field-dependence because of the large coercivity. (**d**) Tunnel magneto-current measured at *V*_*b*_ = 8.7 mV, a bias much greater than the maximum Coulomb blockade threshold voltage of 2*E*_*C*_/*e* ≈ 1 mV, so that the current is gate-independent and the TMR effect can be clearly observed. The magnetic field is swept (indicated by black arrows along the curve) sequentially from *i* to *iv*, with magnetization directions indicated by blue (right electrode), green (center island), and red (left electrode) arrows.

To observe this extra energy cost by a single spin flip, a ferromagnetic single-electron-transistor was designed and constructed. However, the spin diffusion length in ferromagnetic materials is generally too short to sustain spin accumulation^16^. In order to appreciate the magnetocapacitance effect, we used a thin nonmagnetic layer to cover one of the ferromagnetic electrodes. In this way, stable charge dipole is generated inside the nonmagnetic layer. The device, as schematically shown in Fig. 1a, was fabricated using the standard electron-beam lithography technique and two-angle evaporation method. The source and drain electrodes were made of Co, whereas the island was made of 10 nm-thick permalloy (Py, Ni_80%Wt_Fe_20%Wt_), a ferromagnetic material with a smaller coercivity. The Py island was directly covered with a 2 nm-thick aluminum (Al) layer and then a thin tunnel barrier, which was formed by direct evaporation of alumina (Al_2_O_3_) crystal without oxidation process. Since the Al layer is much thinner than ferromagnetic Py layer, its superconductivity is suppressed by the proximity effect^17^,^18^,^19^,^20^. As illustrated in Fig. 1b, the device consists of Co/Al_2_O_3_/Al/Py/Al/Al_2_O_3_/Co with two tunnel junctions in series. The junction area *A* measured about 65 nm × 65 nm, corresponding to a charging energy of the order of 6 K. In addition, a gate-electrode was located about 800 nm away from the island, giving a *C*_*g*_ value of about 0.4 aF and a Coulomb oscillation period of about 0.5 V. All *I-V*_*b*_ characteristics were measured using 4-point probe technique at 120 mK.

The device was symmetrically voltage biased at +*V*_*b*_/2 and −*V*_*b*_/2 on the left and right electrodes, respectively. The magnetic field was applied along the long axis of the cobalt electrodes such that the electrode and island could either be aligned in parallel (P) or AP configuration. Prior to the measurement, the device was subjected to a magnetic field of +50 kOe, to ensure that the magnetization directions of both left and right electrodes and the center island were all parallel to the external field. At this stage, analysis on the conductance height along the edges of Coulomb diamond indicated that the resistance ratio between the left and right junctions (*R*_*left*_/*R*_*right*_) was approximately 1.47. Manipulation of magnetization was then carried out by ramping the magnetic field between ±1000 Oe.

The width of the left electrode was deliberately designed to be narrower so that the magnetization of this electrode remained unchanged due to large shape anisotropy and only magnetizations in the island and right electrode changed direction within this ramping field range. The expected magnetization curve in this field range is illustrated in Fig. 1c, and the measured magneto-current trace depicted in Fig. 1d displays only a single step of suppression in both ramping up and down directions. The plateau appears between 250 Oe and 550 Oe and hence the alignment configuration is well defined and stables at *H*_±_ = ±350 Oe. In terms of alignment configurations between the island and electrodes, there are in total four configurations, which are referred to as All-P, Left-AP, Right-AP, and Both-AP. A careful analysis on the *I-V*_*b*_ characteristics at various gate voltages in these four configurations revealed that the tunneling conductance of both junctions in the AP configuration is reduced by a factor of 1.62 as compared to the P configuration. This TMR effect, together with the difference in left and right junction resistance, results in different current amplitudes in these four configurations. Based on the current values at *H*_±_, we identified the alignment configuration from the largest current as All-P, Left-AP, Right-AP, and Both-AP.

We then took a pause at *H*_±_ in each ramping direction and measured a batch of *I-V*_*b*_ characteristics at varying *V*_*g*_. The charging energy *E*_*C*_ of the device can be deduced directly from the stability diagram (*i.e*. Coulomb blockade diamond) measured in All-P configuration because in this configuration the device can be viewed as a usual non-magnetic SET. The diamond is symmetric in respect to *V*_*b*_ = 0 and *n*_*g*_ ≡ *C*_*g*_*V*_*g*_/*e* = integer point. The *E*_*C*_ is determined to be 510 μ*e*V from the triangular area in both bias polarities. By computing the slopes of the diamond borders, the junction capacitances are determined to be *C*_*Left*_ = 101.8 aF and *C*_*Right*_ = 55.1 aF for the left and right junctions, respectively.

**Figure 2:**
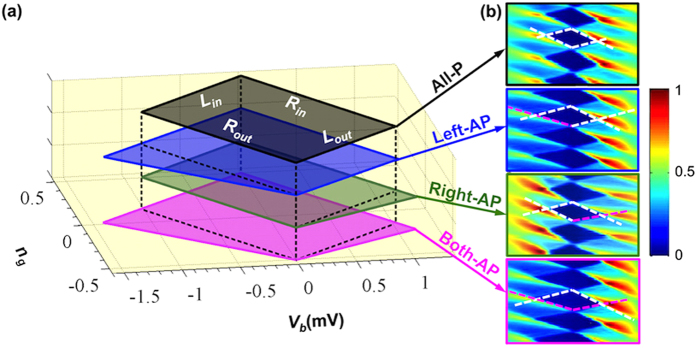
Measured Coulomb diamonds in 4 different alignment configurations. The diamonds are presented in color intensity plots in (**b**) and are stacked together for comparison in (**a**). In (**b**), the dashed lines mark the borders of Coulomb diamonds, and their slopes are used to evaluate effective junction capacitances.

Clear stability diagrams were also obtained for the other three configurations. It is interesting to note that the diamond can become asymmetric in these configurations. To clearly depict the deviation in each alignment configuration, four diamonds are stacked altogether in Fig. 2a. The four borders of these diamonds are indicated by ***L***_***in***_, ***R***_***out***_, ***L***_***out***,_ and ***R***_***in***_. ***L*** and ***R*** stand for tunneling taking place at the left and right junctions, respectively; while the subscription denotes the electron tunneling direction in respect to the island. In the Right-AP configuration the ***L***_***out***_ border tilts clockwise, while in the Left-AP configuration the ***R***_***out***_ border tilts anti-clockwise. In Both-AP, the two borders tilts to overlap with both the ***L***_***out***_ of Right-AP and the ***R***_***out***_ of Left-AP. The change in the border slopes in these three configurations signifies a change in the junction capacitance. We recall that, in an SET, the threshold for electrons to tunnel, *i.e.* the border, through a junction is determined by the capacitance of the counterpart junction. A smaller capacitance corresponds to a more outwardly tilted border. Hence, our observations in Fig. 2 can be summarized as follows: when tunneling *out* from the island through a junction, the electrons experience a decrease in the capacitance of the counterpart junction, *if and only if* the counterpart junction is in AP configuration. This condition of a decrease in the capacitance seen by the tunneling electrons is referred to as the TMC criterion.

In order to characterize quantitatively the diamond asymmetry, we introduce an effective capacitance of Co/Al_2_O_3_/Al/Py, 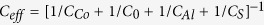. *C*_0_ is simply the intrinsic geometrical capacitance of Al_2_O_3_, given by *C*_0_ = Δ*Q*/Δ*V*, where Δ*V* is the electrical potential difference applied across Al_2_O_3_ and Δ*Q* is the charge induced inside Al_2_O_3_. Note that Δ*Q* is held inside Al_2_O_3_ by an *E*-field given by −Δ*V*/*d*, where *d* is the thickness of Al_2_O_3_. This *E*-field, however, leaks through both Co/Al_2_O_3_ and Al_2_O_3_/Al interfaces, inducing screen charge density *e*Δ*n*(*x*), which in turn damps the leakage *E*-field. As a result, both *e*Δ*n*(*x*) and *V*(*x*) decay exponentially away from interfaces with a characteristic screening length *ξ* of typically 0.5–1 Å^21^.

Despite this decay, the ratio between *e*Δ*n*(*x*) and *dV*/*dx*, remains a constant inside Co and Al. This way, *e*Δ*nAdx*/*dV* inside Co and Al can be, respectively, viewed as 2 serial capacitances, *C*_*Co*_ and *C*_*Al*_, adjacent to *C*_0_. These interfacial capacitances, *C*_*Co*_ and *C*_*Al*_, depend only on their respective electron densities. Lastly, a spin capacitance *C*_*S*_ originated from the TMC effect is used to account for the difference between All-P and other three AP-configurations. To quantify this effect, a TMC ratio, defined as Δ_*TMC*_ = *C*_*eff*_/*C*_*S*_, is introduced. Since *C*_*eff*_ is simply the product of electron charge and slopes of Coulomb diamond borders, it can be readily obtained for each border in all alignment configurations. We further set Δ_*TMC*_ to zero in All-P case, so that Δ_*TMC*_ in the three AP-configurations can be evaluated, and the values are listed in Table 1. From this table, we clearly observe that the junction exhibits substantial Δ_*TMC*_ whenever the magnetocapacitance criterion is fulfilled. The remaining is negligibly small within the error bar. The observed Δ_*TMC*_ value of approximately 40% is the highest among AlO_x_-based magnetic tunnel junctions.

## Discussion

For illustration purpose, we assume that the majority spin in Al is up, denoted by (+) sign, and vice versa. Then, we derive *C*_*S*_ using spin-dependent drift-diffusion model. The detail of this model is provided in the Supplementary Information (S1–S3). It is merely a steady solution to the equation of motion for spin-dependent drifting electrons and back-diffusion electrons inside Al: 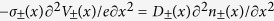, where *x* is the distance from the Al_2_O_3_/Al interface. The drifting and back-diffusion processes are described by the left-hand side and right-hand side of this equation, respectively. *σ*_±_ is conductivity, and *D*_±_ is diffusion coefficient. The chemical potentials 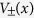
satisfy the spin-dependent Poisson equation, and 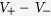 obeys the diffusion-relaxation equation^11^, as described in S1.

Our task is to get a solution for the charge potential 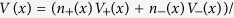 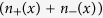 and total charge density perturbation 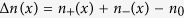; where 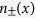 are spin-dependent electron densities, and *n*_0_ is the initial uniform electron density of Al. In All-P configuration, there is no spin accumulation, and the calculated interfacial capacitance is simply the one that incorporates the charge screening at the interface, *i.e*. 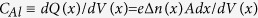. The calculated *C*_*Al*_ is an *x*-independent constant and can serve as a baseline for the calculations of extra serial *C*_*S*_ in the AP-configurations.

**Figure 3:**
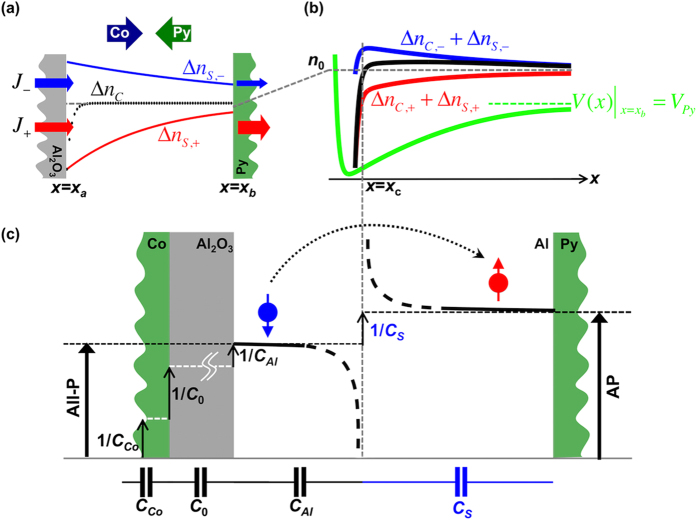
Illustration of charge and spin distributions in the AP-configuration. (**a**) Spin accumulation (and depletion). Blue and green arrows indicate magnetization direction of Co and Py, respectively. Current density *J* is composed of spin up (red) and spin down (blue) components, represented by corresponding colored arrows, whose width indicates the relative magnitude. Dotted black curve resembles the charge density decay in All-P configuration (Figure S1c, S1), and serves as a baseline. Smooth red and blue curves depict spin up depletion and spin down accumulation, respectively. Note that the areas enclosed between the red/blue curve and the equilibrium level *n*_0_ (gray dashed line) are the same. (**b**) A blow-up view of *n*(*x*) and *V*(*x*) around *x*_*c*_, at which *n*(*x*) crosses *n*_0_. Perturbation in *n*(*x*) (black curve) is composed of the spin up (red curve) and spin down (blue curve) contributions. The green curve shows charge potential *V*(*x*) with the block dotted line indicating the height of 
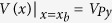
. (**c**) Deduction of inversed capacitance values from *dV*(*x*)/*eΔn*(*x*)*Adx*. All-P plateau (black dash line, left) is elevated to AP plateau (black dash line, right) for 1/*C*_*S*_ after *x*_*c*_. The arrowed sphere (blue and red) represents a single electron spin right after experiencing sequential tunneling from tunnel barrier Al_2_O_3_, whereas the black dotted arrow indicates the flipping event at the cost of *e*^2^/2*C*_*S*_.

In AP-configurations, the current flowing through Al for each spin is not steady (Fig. 3a), giving rise to spin accumulation. This accumulation, in turn, causes a difference between spin-up and spin-down diffusion lengths, *λ*_±_. This difference is taken into account in our finite element analysis described in S2, yielding a solution of 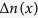
different from that of All-P case. This 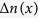 is simply comprised of two spin-dependent components (Fig. 3b), *i*.*e*., 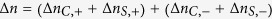, where 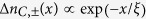 is due to *E*-field penetration alone, which becomes negligible for *x* ≫ 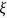, while 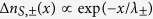 is due to spin-accumulation in AP-configuration. Note that each accumulated/depleted spin diffuses with different spin diffusion length, in contrary to spin-independent charge screening length *ξ*. This difference in *λ* is responsible for the location of *x*_*c*_, where 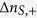 and 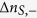 cancel each other out, forming a tiny charge dipole structure. In other words, *x*_*c*_ is just the solution of 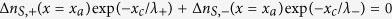, which serves as a critical point across where 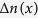 changes sign. In the limit of small current that occurs within Coulomb blockade regime, the difference between *λ*_±_ is small such that the solution for *x*_*c*_ fall within Al thickness, allowing the charge dipole to exist inside Al with 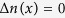 at *x* = *x*_*c*_. Because of the finite 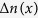
value at *x* > *x*_*c*_, an extra serial capacitance *C*_*S*_ in AP-configuration is generated, which can be calculated using 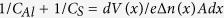, as shown in Fig. 3c. Note that the calculated 1/*C*_*S*_ is only dependent on the magnetic configuration of our device since 1/C_*Al*_ is used as a reference for 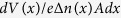 calculation. The induced tiny charge dipole, having an equivalent capacitance *C*_*S*_, thus acts as a serial capacitance to *C*_*Al*_ in the AP-configuration. The existence of this charge dipole requires that *x*_*c*_ be greater than but close to the screening length *ξ*. While the lower limit of Al thickness is set by *x*_*c*_, the upper limit is set by the spin diffusion length λ and the effect of exchange proximity (see S1). Since it is a single electron sequential tunneling process, this implies an extra cost of “charging energy” for a single spin flip event, which takes place in AP-configuration. The positive side of the charge dipole is close to the Al_2_O_3_ interface, regardless of the current direction, as explained in S3. However, if the electrons flow in opposite direction to the one shown in Fig. 3a, *i*.*e*. from Py island towards Al_2_O_3_/Al interface, the charge accumulates from Al_2_O_3_/Al interface and compensates the positive side of the charge dipole. This would wash away the charge dipole, and the TMC diminishes, causing Coulomb diamond to display asymmetry with respect to the bias direction of *V*_*b*_.

To conclude, we observed and verified the TMC effect in a ferromagnetic SET based on capacitance values determined from the asymmetry of Coulomb diamonds. The asymmetry reflects a decrease in the capacitance value of an AP-aligned junction through which electrons tunnel into the island. This decrease is quantitatively described by spin-dependent drift-diffusion model. In AP configurations, spin-accumulation causes a difference between spin-up and spin-down diffusion lengths that provides a ground for the creation of a tiny charge dipole. This charge dipole acts as an extra serial capacitance that gives rise to the observed TMC effect. The magnetocapacitance also implies an extra energy threshold for a single spin to flip whenever it is entering the island through an AP-aligned tunnel junction.

## Additional Information

**How to cite this article**: Lee, T. H. and Chen, C. D. Probing Spin Accumulation induced Magnetocapacitance in a Single Electron Transistor. *Sci. Rep.* **5**, 13704; doi: 10.1038/srep13704 (2015).

## Supplementary Material

Supplementary Information

## Figures and Tables

**Table 1:** Δ_*TMC*_ in percentage (%) calculated for each of four borders of Coulomb diamonds in the three configurations that exhibit asymmetric diamond.

Configuration	*L*_*in*_	*R*_*out*_	*L*_*out*_	*R*_*in*_
Left-AP	2.4	***40.3***	3.2	0.7
Right-AP	−0.9	0.6	***31.0***	2.9
Both-AP	1.2	***41.8***	***30.3***	1.8

All values are within an error bar of 4%.
