# Supplementary material for: Probing Spin Accumulation induced Magnetocapacitance in a Single Electron Transistor
Source: Sci Rep. 2015 Sep 8;5:13704. doi: 10.1038/srep13704 (PMC4562261; doi:10.1038/srep13704)
Supplement: Supplementary Information [file srep13704-s1.pdf]

Supplementary Information for "Probing Spin Accumulation induced Magnetocapacitance in a Single Electron Transistor"

Teik-Hui Lee[1,2,3] and Chii-Dong Chen[3]

[1]*Department of Physics, National Taiwan University, Taipei 106, Taiwan*

[2]*Nano Science and Technology Program, Taiwan International Graduate Program, Academia Sinica, Taipei 115, Taiwan*

[3]*Institute of Physics, Academia Sinica, Taipei 115, Taiwan*

S1. Spin-dependent drift-diffusion model:

At any instance of time t and any location x inside Al layer, we have the spin-dependent electron densities, $n_{\pm}(x,t)$, and chemical potentials $V_{\pm}(x,t)$. At the Al/Py interface (x=x_b), the spin polarization of Al, $P(x,t)=\left[n_{+}(x,t)-n_{-}(x,t)\right]/\left[n_{+}(x,t)+n_{-}(x,t)\right]$ is exchange-induced by the much thicker Py as a result of proximity effect [1,2]. Therefore, the initial value for $P(x,t)$ is non-zero, spatial-independent, and takes up a value close to the one found in Py, *i.e.*, $P_0 \approx P_{Py}$. At x=x_a, there is a barrier interface Al_2O_3/Al where electrons tunnel into Al from Co, giving rise to spin-dependent current density, which can be estimated based on Jullière model:

$$J_{\pm}\left(x=x_a\right)=J\frac{\left[\left(1\pm P_{Co}\right)\left(1\pm P_0\right)\right]}{\left[\left(1+P_{Co}\right)\left(1+P_0\right)+\left(1-P_{Co}\right)\left(1-P_0\right)\right]} \qquad (1)$$

where P_{Co} is spin polarization of Co, and J=-I/A

In All-P configuration, we can assume $P_{Co}=P_0$. The spin-dependent but spatial-independent current density is $J_{\pm}=J\left(1\pm P_0\right)/2$, and thus there is no spin-accumulation. On the other hand, there is charge screening, which in All-P configuration is calculated below. First, the boundary condition of the spin-dependent E-field (gradient of $V_{\pm}(x,t)$) is determined such that $\partial V_{+}/\partial x=\partial V_{-}/\partial x=-\Delta V/d$ is a constant at x=x_a, while vanishes

at x=x_b (Figure S1a). The latter is set under the assumption that the electric field vanishes at the Al/Py interface. We then consider the spin-dependent driven-drift current caused by the boundary E-field, *i.e.*, $J_{ext,\pm}(x,t) = -\sigma_\pm(x,t)\partial V_\pm(x,t)/\partial x$, with spin-dependent conductivity of Al, $\sigma_\pm(x,t) = 0.5[1 \pm P(x,t)]\sigma_{Al}$, where σ_{Al} is the intrinsic conductivity of Al. Plugging this in the continuity equation we obtain spin-dependent depletion causing by external E-field after an infinitesimal time dt:

$$dn_{ext,\pm}(x,t) = -\sigma_\pm(x,t)\frac{\partial^2 V_\pm(x,t)}{e\partial x^2}dt \tag{2}$$

This $dn_{ext,\pm}(x,t)$ yields a deviation $\delta_\pm(x,t)dn_{ext,\pm}(x,t)$ from equilibrium Fermi level, where $\delta_\pm(x,t) \propto n_\pm^{-1/3}(x,t)$ is the level spacing inside Al. As a result, a steady back-diffusion current is established, *i.e.*, $J_{diff,\pm}(x,t) = -\sigma_\pm(x,t)\delta_\pm(x,t)\partial n_\pm(x,t)/\partial x$. Using again the continuity equation, we obtain the refill caused by internal back-diffusion for each spin channel:

$$dn_{diff,\pm}(x,t) = D_\pm(x,t)\frac{\partial^2 n'_{m,\pm}(x,t)}{\partial x^2}dt \tag{3}$$

, where $D_\pm(x,t) = \delta_\pm(x,t)\sigma_\pm(x,t)/e \propto n_\pm^{2/3}(x,t)$ is the spin-dependent diffusion coefficient. Summing up contributions from $dn_{ext,\pm}(x,t)$ and $dn_{diff,\pm}(x,t)$ after each dt, the new density distribution for each spin is $n_\pm(x,t+dt) = n_\pm(x,t) - dn_{ext,\pm}(x,t) + dn_{diff,\pm}(x,t)$, as shown in Figure S1b. This deviation from the initial uniform charge density $n_{\pm,0}$, *i.e.*, $e[n_\pm(x,t) - n_{\pm,0}]$, modifies the chemical potential profile $V_\pm(x,t)$, as described by the spin-dependent Poisson's equation:

$$\frac{\partial^2}{\partial x^2}\left[\frac{1}{2}(1 \pm P(x,t))V_\pm(x,t)\right] = \frac{e[n_\pm(x,t) - n_\pm(x,0)]}{\varepsilon_0} \tag{4}$$

, where ε_0 is free space electric permittivity. At this point $V_{\pm}(x,t)$ can be solved and plugged back in equation (2) to form a complete iteration loop. We note that, in All-P configuration, even though spin-up and spin-down charge densities are different, but due to nonzero $P(x,t)$, the chemical potential for spin-up, and spin-down are the same at all instants, *i.e.* $V_{+}(x,t)=V_{-}(x,t)$.

In addition, the average chemical potential $V(x,t)=V_{+}(x,t)\left[1+P(x,t)\right]/2+V_{-}(x,t)\left[1-P(x,t)\right]/2$ is spin-independent. Upon convergence, the sum $dn_{+}(x,t)/dt+dn_{-}(x,t)/dt=0$ is accomplished, giving time-independent $V(x)$ and $n_{\pm}(x)$ distributions. The total charge density perturbation is then given by

$$\Delta n(x)=n_{+}(x)+n_{-}(x)-n_0 \qquad (5)$$

Where $n_0=n_{+,0}+n_{-,0}$. Accordingly, the interfacial capacitance $C_{Al}=e\Delta n(x)Adx/dV(x)$ can be calculated.

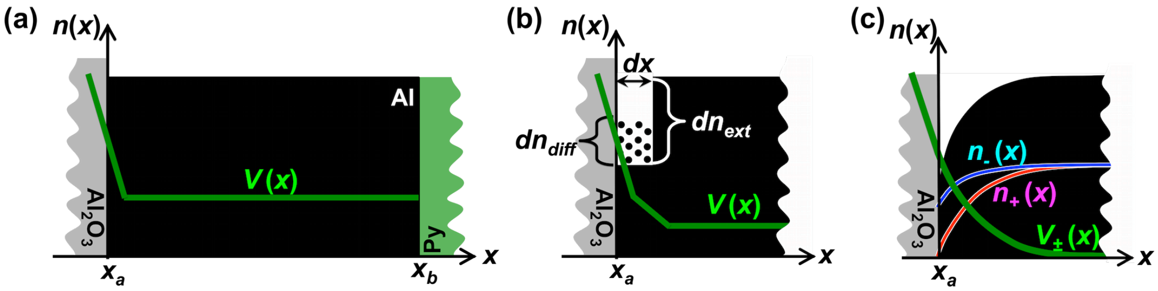

Figure S1 (color online) | Charge screening process in All-P configuration. (a) Initial $n(x)$ and $V(x)$ profiles. The dark area shows initial uniform free electron density inside Al and the green line shows initial $V(x)$ at t=0. (b) $n(x)$ and $V(x)$ profiles after the first iteration. Scooped area represents depleted density $dn_{\text{ext}}(x)$ and dotted area shows refilled back-diffusion density $dn_{diff}(x)$. (c) Saturated $n(x)$ and $V(x)$. Both curves have exponential form with a characteristic length x. Red and blue curve inside dark area represent the spin up and down components of $n(x)$, respectively.

Note that both $V(x)$ and $\Delta n(x)$ follow an exponential decay with the screening length *x*, as depicted in Figure S1c, but the calculated C_{Al} is constant everywhere inside Al. This screening length *x* turns out to be the same as that for the case of non-magnetic junctions[3], in which $P(x,t)=0$, and $n_+(x)=n_-(x)$, $V_+(x)=V_-(x)$. The calculated C_{Al} serves as a baseline for the calculations of extra C_S in the AP-configurations.

S2. Spin-dependent diffusion lengths

In AP-configuration, we assume $P_{Co}=-P_0$, and equation (1) is reduced to $J_\pm(x=x_a)=J/2$ (Figure 3a in *Main Text*). Consequently, the spin dependent current density obeys diffusion equation, which in steady state is given as:

$$dJ_\pm(x,t)=D_\pm(x,t)\left[\frac{\partial^2\left(J_\pm(x,t)-J_\pm(x,0)\right)}{\partial x^2}-\frac{J_\pm(x,t)-J_\pm(x,0)}{\lambda_\pm^2}\right]dt \qquad (6)$$

After each *dt*, we have $J_\pm(x,t+dt)=J_\pm(x,t)+dJ_\pm(x,t)$, and the accumulated/depleted spin density is [4-6]:

$$dn_{acc,\pm}(x,t)=\frac{\partial J_\pm(x,t)}{e\partial x}dt \qquad (7)$$

This spin-accumulation causes a difference in the chemical potential for spin-up and spin-down electrons. This difference in turn triggers spin flips and that produces a "refill" of spin density:

$$dn_{flip,\pm}(x,t)=\left[3\left(1\mp P(x,t)\right)D_\pm(x,t)\frac{n_\pm(x,t)\delta_\pm(x,t)-n_\mp(x,t)\delta_\mp(x,t)}{4\delta_\pm(x,t)\lambda_\pm{}^2}\right]dt \qquad (8)$$

, where $n_{\pm}(x,t)\delta_{\pm}(x,t)-n_{\mp}(x,t)\delta_{\mp}(x,t)$ is the chemical potential difference. In the mean time, spin accumulation also cause a difference in diffusion coefficient for each spin, $D_{\pm}(x,t)\propto n_{\pm}^{2/3}(x,t)$, which depends on their respective chemical potential. As a result, each spin has a different diffusion length, since $\lambda_{\pm}\propto\sqrt{D_{\pm}}$. Following Eq. (2) and (3), the total spin density at *t=t+dt* is the sum of all contributions, *i.e.*,

$$n_{\pm}(x,t+dt)=n_{\pm}(x,t)-dn_{ext,\pm}(x,t)+dn_{diff,\pm}(x,t)+dn_{acc,\pm}(x,t)-dn_{flip,\pm}(x,t) \qquad (9)$$

This is then plugged back in Eq. (4) to complete the new iteration loop. Finally, upon convergence, the system reaches a steady state so that the sums $dn_{\pm}(x,t)/dt-dn_{\mp}(x,t)/dt=0$ and $dn_{+}(x,t)/dt+dn_{-}(x,t)/dt=0$ give the new time-independent $V_{\pm}(x)$ and $n_{\pm}(x)$ distributions. By updating Eq. (5), we deduced C_S in AP-configuration (in the *Main Text*).

S3. Formation of Charge dipole by Spin Accumulation

Our device is composed of tunnel junctions that comprise of structure as follow: $Co/Al_2O_3/Al/Py$. Since Al is located between magnetically aligned Co and Py, it serves as an ideal place to conceive spin accumulation in AP configuration. The current flowing through tunnel barrier obeys Julliere's model as in Eq. (1). Hence in AP configuration, current for spin up and spin down are in same proportion to each other. Since Al is exchange induced in Ohmic proximity by Py, the current inside Al layer is initially uniformly polarized. As a result, for each spin, there is a discrepancy between tunneling current at Al_2O_3/Al interface and the current inside Al. As in Eq. (6), due to this discrepancy, the current for each spin diffuse away from the Al_2O_3/Al interface towards Py. This non-steady of the flowing current, manifested as the gradient of the current density for each spin as shown in Eq. (7), causing each spin to either accumulate or deplete depending on the flowing direction. Spin-down (blue) is accumulated when electrons flow from Al_2O_3/Al interface towards Py (Figure S2a), while spin-up (red) is

accumulated when electrons flow otherwise (Figure S2b). As electron density determine diffusion coefficient, thus the spin component that is accumulating has a longer diffusion length as compared to the one that is depleting. And the total accumulated spin across Al must equate the total depleted spin, due to the conservation of charge.

$$\int_{x_a}^{x_b} \Delta n_{S,+}(x)\, dx = \int_{x_a}^{x_b} \Delta n_{S,-}(x)\, dx \tag{10}$$

Where $\Delta n_{S,\pm}(x) = \int_0^\infty \frac{\partial J_\pm(x,t)}{e\partial x} dt$ is the accumulated/depleted amount of spin due to non-steady current of each spin. Consequently, the accumulating spin is less than the depleting spin at Al_2O_3/Al interface, while otherwise at Al/Py interface, thus forming a charge dipole within Al (Figure S2c). Note that the direction of the created charge dipole is independent of the electron flowing direction.

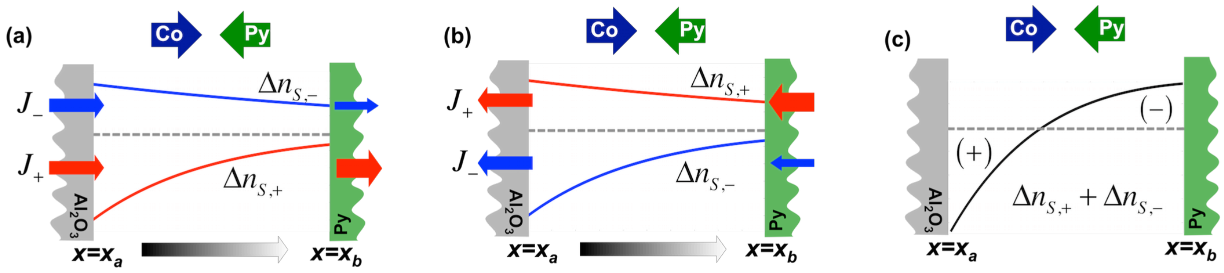

Figure S2 (color online) | Charge dipole formation inside Al layer due to spin accumulation. Grey line indicates intrinsic uniform electron density. Note that diffusion (gradient grey arrow at bottom) always diffuse away from tunnel barrier. (a) Electrons flow from left to right. Current density J is composed of spin up (red) and spin down (blue) components, represented by corresponding colored arrows, whose width indicates the relative magnitude. (b) Electrons flow from right to left. (c) Charge dipole as the summation of spin-up and spin-down electron densities

S4. Numeric calculation of Δ_{TMC} value

Within our model, the Δ_{TMC} value increases with the spin diffusion length λ. For λ→0, there is no spin accumulation and the All-P case is recovered so that Δ_{TMC}→0. Contrarily, in the case of λ→∞, it is not possible to flip spins, and the energy required to flip a single spin ($e^2/2C_S$ in Fig. 3c) becomes infinite so that Δ_{TMC}→100%. In the numeric calculations, due to some technique issues (such as limited memory and CPU speed), we could only get a Δ_{TMC} value of up to 80%. The calculated Δ_{TMC} as a function of spin diffusion length λ using the experimental device parameters and the reported polarization values[7] for Co (35%) and Py (40%) is shown in Fig. S3. It is found that, for a given λ, the Δ_{TMC} value is insensitive to the magnitude of tunneling current and the polarization values. However, there are factors such as interfacial roughness and impurities in Al that are not considered in the present calculation. For the observed $\Delta_{TMC} \approx 40\%$, this calculation implies a spin diffusion length of about 2.8nm, and the dipole location x_c, which is related to the spin diffusion length, is calculated to be about 1.2nm.

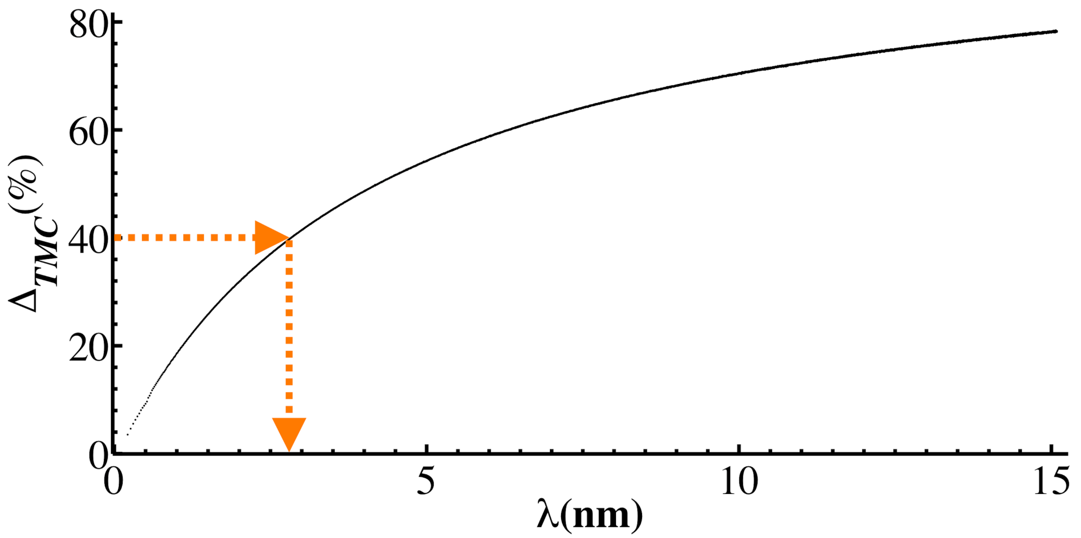

Figure S3 (color online) | Calculated Δ_{TMC} value as a function of spin diffusion length λ in Al layer under AP-configuration. Orange dash arrow corresponds to the experimentally observed Δ_{TMC}, suggesting a spin diffusion length of 2.8nm.

References

1. Mitsuzuka, T., Matsuda, K., Kamijo, A. & Tsuge, H. Interface structures and magnetoresistance in magnetic tunnel junctions. *Journal of Applied Physics* **85,** 5807 (1999).
2. Moodera, J., Taylor, M. & Meservey, R. Exchange-induced spin polarization of conduction electrons in paramagnetic metals. *Physical Review B* **40,** 11980–11982 (1989).
3. Ku, H. Y. & Ullman, F. G. Capacitance of Thin Dielectric Structures. *Journal of Applied Physics* **35,** 265(1–3) (1964).
4. Johnson, M. Spin accumulation in gold films. *Physical Review Letters* **70,** 2142 (1993).
5. Zaffalon, M. & van Wees, B. Spin injection, accumulation, and precession in a mesoscopic nonmagnetic metal island. *Physical Review B* **71,** 125401 (2005).
6. Jedema, F., Nijboer, M. & et al. Spin injection and spin accumulation in all-metal mesoscopic spin valves. *Physical Review B* **67,** 085319 (2003).
7. Meservey, R. & Tedrow, P. M. Spin-polarized electron tunneling. *Physics Reports* **238,** 173–243 (1994).
